# Role of Vitamin K in Intestinal Health

**DOI:** 10.3389/fimmu.2021.791565

**Published:** 2022-01-05

**Authors:** Yujiao Lai, Hori Masatoshi, Yanbo Ma, Yuming Guo, Bingkun Zhang

**Affiliations:** ^1^ State Key Laboratory of Animal Nutrition, College of Animal Science and Technology, China Agricultural University, Beijing, China; ^2^ Department of Veterinary Pharmacology, Graduate School of Agricultural and Life Sciences, The University of Tokyo, Tokyo, Japan; ^3^ Department of Animal Physiology, College of Animal Science and Technology, Henan University of Science and Technology, Luoyang, China

**Keywords:** VK, intestinal health, VKDPs, IBDs, intestinal disease

## Abstract

Intestinal diseases, such as inflammatory bowel diseases (IBDs) and colorectal cancer (CRC) generally characterized by clinical symptoms, including malabsorption, intestinal dysfunction, injury, and microbiome imbalance, as well as certain secondary intestinal disease complications, continue to be serious public health problems worldwide. The role of vitamin K (VK) on intestinal health has drawn growing interest in recent years. In addition to its role in blood coagulation and bone health, several investigations continue to explore the role of VK as an emerging novel biological compound with the potential function of improving intestinal health. This study aims to present a thorough review on the bacterial sources, intestinal absorption, uptake of VK, and VK deficiency in patients with intestinal diseases, with emphasis on the effect of VK supplementation on immunity, anti-inflammation, intestinal microbes and its metabolites, antioxidation, and coagulation, and promoting epithelial development. Besides, VK-dependent proteins (VKDPs) are another crucial mechanism for VK to exert a gastroprotection role for their functions of anti-inflammation, immunomodulation, and anti-tumorigenesis. In summary, published studies preliminarily show that VK presents a beneficial effect on intestinal health and may be used as a therapeutic drug to prevent/treat intestinal diseases, but the specific mechanism of VK in intestinal health has yet to be elucidated.

## Introduction

**Figure 1:**
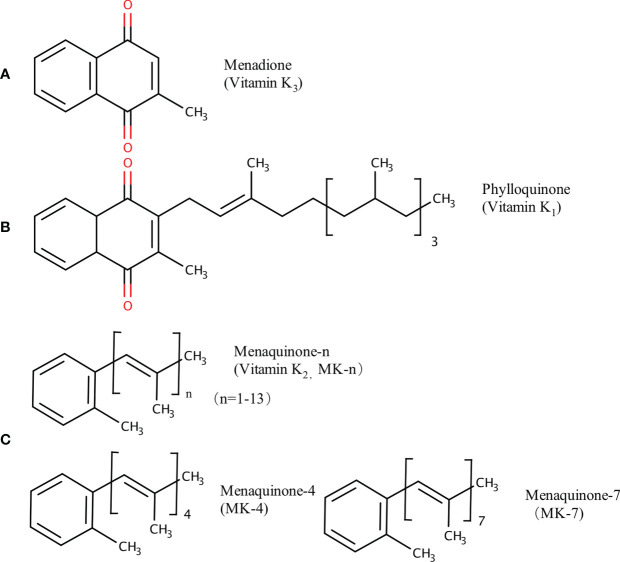
Chemical structures of VK compounds: **(A)** 2-methyl-1,4-naphthoquinone (menadione, K_3_), **(B)** 2-methyl-3-phytyl-1,4-naphthoquinone (phylloquinone, K_1_), and **(C)** when *n* = 4 and 7, 2-methyl-3-geranyl-geranyl-1,4-naphthoquinone (menaquinone-4, MK-4) and 2-methyl-3-all-trans-farnesyldigeranyl-1,4-naphthoquinone (menaquinone-7, MK-7) are the two common forms of menaquinones (VK_2_). The figure is in non-editable format.

**Figure 2:**
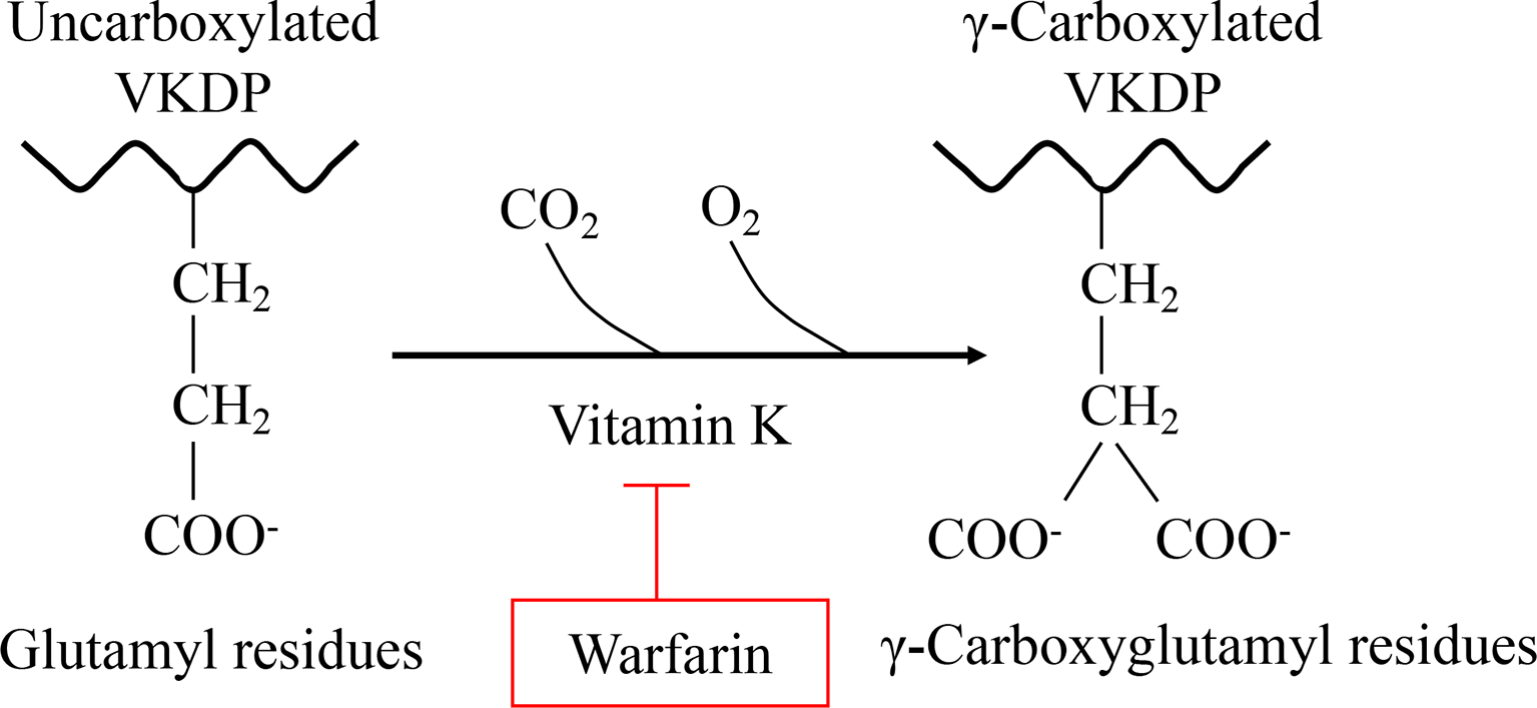
VK is essential for the formation of Gla. Gla, a unique amino acid, is produced by a VK-dependent posttranslational modification of Glu in all Gla-containing proteins. This carboxylation process can be inhibited by warfarin.

**Figure 3:**
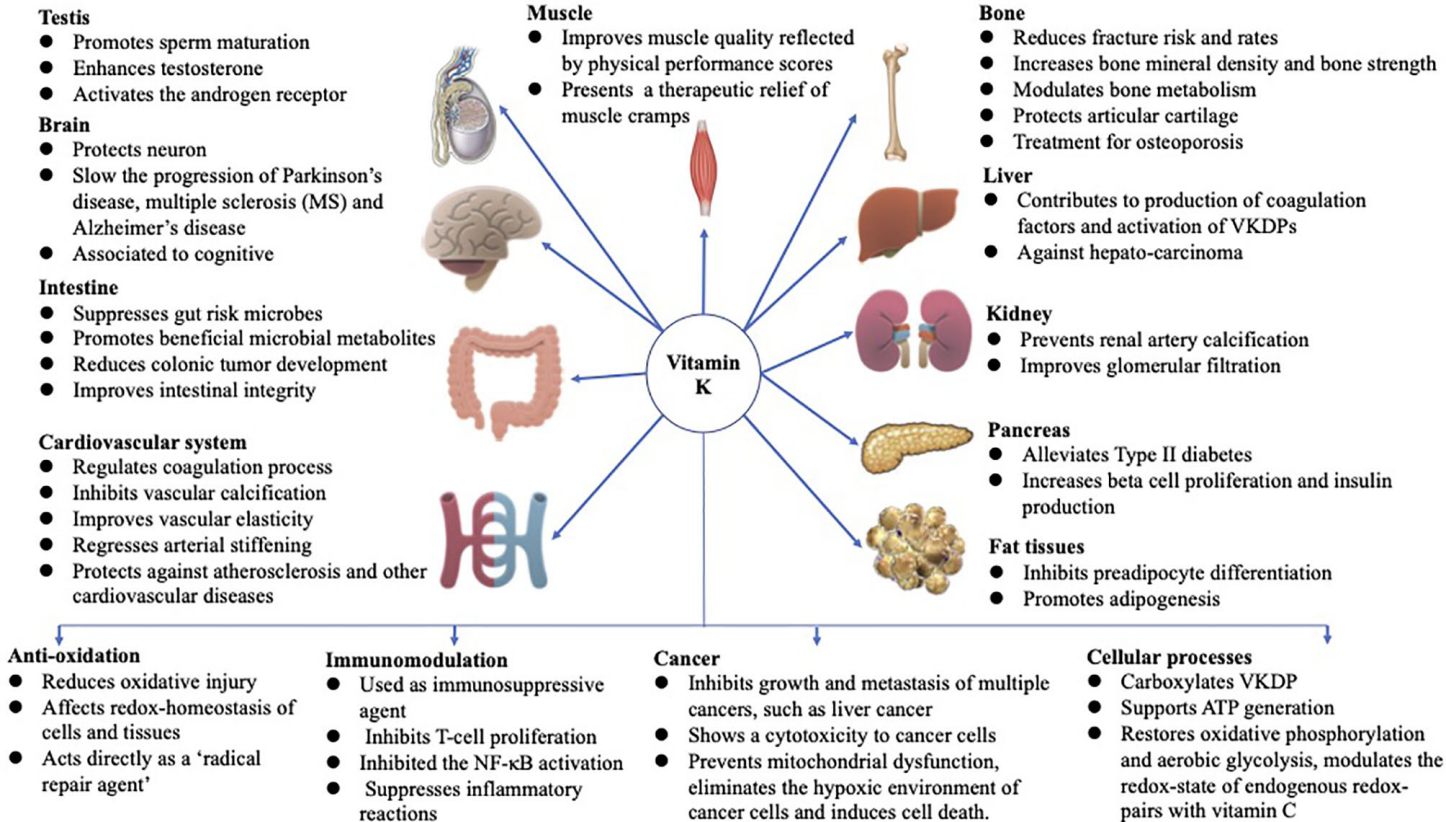
Functions of VK in multiple-organ systems, such as testis (9), brain (10–14), intestine (15–17), muscle (18, 19), bone (20–22), liver (7, 23), kidney (24, 25), pancreas (26, 27), fat tissues (28–30), and cardiovascular system (31–34), and biological processes involved in anti-oxidation (3, 4), immune response and anti-inflammation (5, 6), and cancer progression (7, 8), and associated with protective and promoting roles in diverse organs or tissues throughout the human body are summarized above. The figure is in a non-editable format.

Vitamin K (VK), a fat-soluble factor, is a generic term for a series of structurally related compounds (1), which shares a common ring structure of 2-methyl-1,4-naphthoquinone. However, forms of VK differ in the degree of saturation and the varying lengths of the aliphatic side chain attached to the 3-position (**Figure 1**). VK is an essential lipid-soluble vitamin that functions as a cofactor for γ-glutamyl carboxylase (GGCX) which is an integral membrane protein and catalyzes the conversion of glutamate (Glu) residues into γ-carboxyglutamate (Gla) essentially and enables VKDPs to perform their biological functions (2). This biological process is inhibited by warfarin (**Figure 2**). In addition to the well-known biological function of blood coagulation and bone metabolism, emerging studies support VK involved in multiple cellular and physiological processes such as oxidative stress (3, 4), immune response and anti-inflammation (5, 6), and cancer progression (7, 8) and associated with protective and promoting roles in diverse organs or tissues, such as testis (9), brain (10–14), intestine (15–17), muscle (18, 19), bone (20–22), liver (7, 23), kidney (24, 25), pancreas (26, 27), fat tissues (28–30), and cardiovascular system (31–34) (**Figure 3**).

The intestinal tract is the primary organ responsible for the digestion and absorption of nutrients. Also, the intestinal system combats invasive compounds with the help of defense mechanisms such as detoxification activities and the immune system. Factors, such as nutrition, gut environment, physiological status, and the microbial compositions, are likely to modulate the functionalities of the intestine. Therefore, any impairment in gut integrity may lead to enteritis, for example, inflammatory bowel diseases (IBDs). IBDs, comprising both ulcerative colitis (UC) and Crohn’s disease (CD), are lifelong, chronic, immunologically inflammatory disorders of the gastrointestinal tract. It occurs as a result of altered interactions between the mucosal immune system and gut bacteria (35). The incidence of IBDs is about 1-3 in 1,000 individuals (36). Typical symptoms of IBDs include diarrhea, abdominal pain, and rectal bleeding (37), which are common worldwide, especially in western countries (38). Besides, IBDs can increase the risk of colorectal cancer (CRC), which is the third leading cause of malignant tumors (39). The aberrant immune response to gut microbes is thought to result in IBDs in genetically susceptible individuals. The host is susceptible to colonization by pathobionts resulting from functional and compositional dysbiosis of the gut microbiome. In addition, oxidative stress exerts a critical effect on the initiation and occurrence of relapses in UC (40). Therapeutic approaches, such as the regulation of interactions between the gut bacteria and the immune system, are used to restore intestinal homeostasis or reduce inflammation. In addition, when UC is in the active stage and on remission of the disease, malnutrition accounts for about 85% of patients with IBD (41). Micronutrient deficiencies, such as deficiency in VK, vitamin D, iron, selenium, zinc, folic acid, and vitamin B_1_, B_6_, or B_12_, have also been recorded in more than half of patients with IBD (41). Administration of micronutrients therefore seems to be a novel therapeutic approach to alleviate intestinal diseases, particularly those that can relieve inflammation, reduce oxidation, and inhibit invasion of pathogenic bacteria. As a micronutrient, emerging evidence on the immunoregulatory effect of VK in intestinal health suggests novel roles for VK in gut disease health and beyond the VK typical function in hemostasis (13, 32, 42, 43).

Previous studies demonstrated that VK reduced interleukin (IL)-6 in a murine model of colitis (44); improved the antioxidant capabilities (45); improved intestinal bacteria flora (15); improved intestinal alkaline phosphatase (IAP) (46), and adiponectin (ADPN), the nuclear receptor vitamin D receptor (VDR), and the adenosine 5′-monophosphate (AMP)-activated protein kinase (AMPK) activity (15); contributed to blood coagulation in gastrointestinal bleeding (GIB) (47); and alleviated IBD (16, 44) and CRC (15). Thus, gathering and summarizing the latest findings on VK actions in the intestine other than coagulation is important and should be summarized and elucidated by studies from laboratories. The present study focuses on the relationship between VK, intestinal health, and the mechanisms through which VK modulates intestinal microbes, exerts anti-inflammatory and antioxidant effects, and improves intestinal function.

## Various Sources of VK

VK comes from natural sources and chemical synthesis (menadione, also known as VK_3_). Natural VK exists mainly in two biologically active forms: vitamin K_1_ (phylloquinone, also called K_1_) is present in plant margarine and vegetables (48) which is the major dietary source of VK in the US diet (49). Vitamin K_2_ (K_2_) consists of a group of menaquinones (MK-n, varies from MK-4 to MK-13) is present in natto, egg yolk, meat, liver, cheese, curd cheese, and butter (48) and biosynthesized by gut bacteria (50). Among all menaquinones, MK-4 and MK-7 are the most well-studied. Information on a detailed content and adequate intake of VK in natural sources was provided in a recent review (51, 52). The total VK dietary intake comprises K_1_, MK-4, and MK-7 (more than 60%, 24%, and 7%, respectively) (53). In animals and human beings, MK-4 is catabolized from K_1_ with K_3_ as an intermediate with UbiA prenyltransferase domain-containing 1 (UBIAD1) (54), and partially from long-chain MKs in extrahepatic tissues, for example, salivary gland, brain, pancreas, reproductive organs, kidney, and fat (1). However, when K_1_ isoprenoids are derived from mevalonate, merely 5%–25% of K_1_ intake is converted into MK-4, followed by the synthesis of other MKs in some but not all tissues *via* prenylation (55). The prenylation process seems to happen independently from intestinal bacteria (56, 57).

Apart from the dietary intake sources, MKs are mainly synthesized by gut microbiota, predominantly in the ileum (58). MKs are abundant in the human gut, and the concentrations of different MK forms within the intestine show considerable intraindividual and interindividual variations related to heterogeneity in the intestinal microbiome composition (59). Bacteria can release MKs in lipid-soluble (60) or other forms of complexes, such as short-chain quinones (61). The major forms of MK-6 are synthesized by *Eubacterium lentum*, MK-7 by *Veillonella*, MK-8 by *Escherichia coli*, and MK-10 and MK-11 by *Bacteroides* species (50, 62). However, the disparity in fecal VK content is not owing to differences in the principal dietary VK forms (i.e., K_1_ and MK-4), but it is based on discrepancies in the fecal content of some bacterially derived MKs (63). The intestinal bacteria are capable of producing MKs, yet information on the bioavailability of this intestinal MK supply is limited. Majority of these MKs are bound to bacterial membranes present in the gut (1). Previous studies showed that bioactivity and bioavailability differed across vitamers (64–66), with evidence approving superior bioavailability, higher bioactivity, and probably unique functions of some bacterially synthesized MK forms rather than K_1_ (67–69). Even though gut bacteria synthesize a great deal of MKs, the bioavailability of bacterial menaquinone is bad in general, and diet is the principal source of functionally available K_2_ (55, 70). There are studies showing that a short-term decrease in dietary VK intake is not compensated by gut bacteria synthesized MKs (71, 72). Actually, inadequate dietary intake (73), restorative proctocolectomy (74), IBD (75), liver dysfunction (76). chronic kidney disease (CKD) (77, 78), and antibiotic administration (79) could cause a VK-deficient state.

## Intestinal Absorption and Metabolism of VK

**Figure 4:**
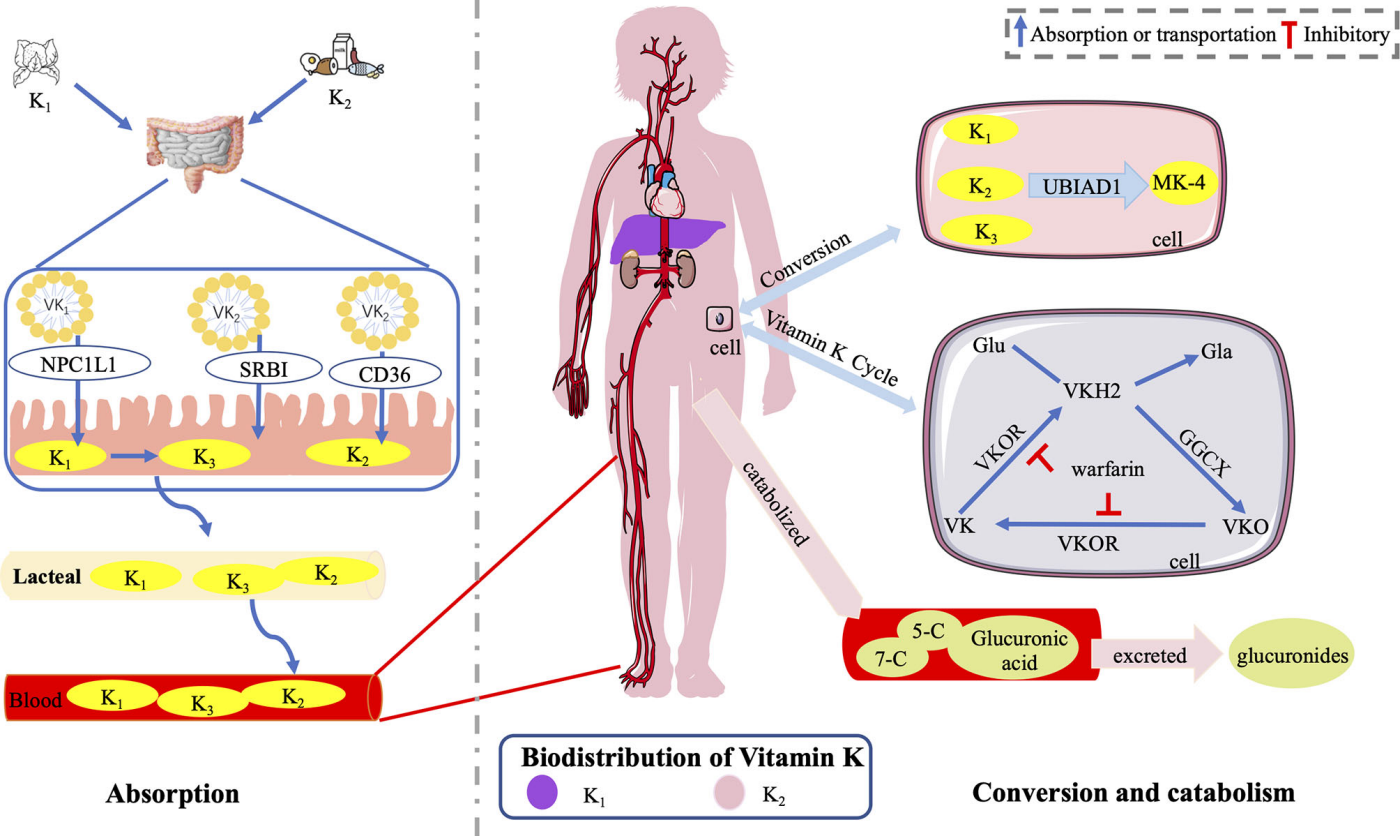
Absorption, distribution, and catabolism of VK. Once the dietary VK reaches the intestinal lumen, it is absorbed into mixed micelles through the NPCIL1 protein, SR-BI, and CD 36. Mixed micelles are absorbed by small-intestinal enterocytes, incorporated into CM, and secreted from gut villi into lacteals. Then, they join the larger lymphatic vessels where they are released through the thoracic duct into the bloodstream. K_1_ is converted into K_3_ in the gut, delivered to tissues, and subsequently converted into MK-4 with UBIAD1. K_1_ is retained in the liver, while K_2_ is redistributed to the circulation and (extra-)hepatic tissues (51). VK epoxide cycle involving GGCX and VKOR, which is responsible for VK regeneration by converting into VK, VKH_2_, and VKO. In humans, the polyisoprenoid side chain of VK is catabolized into two major carboxylic acid metabolites of 7- and 5-carbon side chains. Then, after glucuronic acid conjugation, it is finally excreted as glucuronides in the bile and urine primarily. The figure is in a non-editable format.

Intestinal absorption of VK involves bile salt- and pancreatic-dependent solubilization. Once the dietary VK reaches the intestinal lumen, it is absorbed into a mixture of bile salts, pancreatic lipolysis products, and other dietary lipids (80). Mixed micelles are absorbed by small-intestinal enterocytes and incorporated into nascent chylomicron (CM). At the same time, they are secreted from gut villi by exocytosis into the lymphatic capillaries (lacteals) through the proximal intestine (81) and then join the larger lymphatic vessels where they are released through the thoracic duct into the bloodstream (80). CM enters the capillary layer of peripheral tissues in the bloodstream, where it loses much of its triglyceride (TG)-producing chylomicron remnant (CR) through the action of lipoprotein lipase. The formed CR has a centralized lipid core, and only a small quantity eventually reenters the circulatory system (80) (**Figure 4**).

The Niemann–Pick C1-like 1 (NPCIL1) protein, the scavenger receptor class B-type I (SR-BI), and the cluster-determinant 36 (CD 36) are thought important for intestinal VK absorption (82). NPCIL1 is a primary importer for K_1_ in the gut, while the physiological role and significance of SR-BI and CD 36 as importers for K_1_ in the small bowel need further studies (82). The absorption of dietary VK is slower than that of pure K_1_ due to different matrices (serum peak values at 6 vs. 4 h after ingestion) (83). Different forms of VK are transported by different carriers. TG-rich lipoproteins transport K_1_ predominantly, while low-density lipoproteins transport long-chain MKs primarily in the postprandial state (55). In terms of K_2_, MK-4, MK-5, and MK-6 may be most effective in nature (65). MK-7 to MK-13, which are synthesized by gut bacteria, are not efficiently absorbed with relatively increasing longer isoprene units (65).

The distribution of VK in the body organ tissue is inconsistent; K_1_ was found mainly distributed in the liver, while K_2_ was present in the extrahepatic tissues at higher levels (84). Besides, the concentrations of K_1_, MK-4, and some long-chain MKs presented sex-specific differences in rat tissues (liver, kidney, brain, mesenteric adipose tissue, and pancreas) in response to the dietary K_1_ levels (85).

The dietary K_1_ was converted into K_3_ in the gut, delivered to tissues, and subsequently converted into MK-4 with UBIAD1 (54, 86). *In vitro*, both K_1_ and K_2_ were rapidly metabolized into a mixture of quinone, hydroquinone, and epoxide (87). In humans, K_1_ and MKs are catabolized in the liver and excreted sharing a common degradative pathway. Initially, the polyisoprenoid side chain of VK is catabolized into two major carboxylic acid metabolites of 7-carbon [2-methyl-3-(5-carboxy-3′-methyl-2′-pentenyl)-1,4-naphthoquinone] and 5-carbon side chains [2-methyl-3-(3′-3′-carboxymethylpropyl)-1,4-naphthoquinone]. Then, after glucuronic acid conjugation, it is finally excreted as glucuronides in the bile and urine primarily (55, 88, 89).

The metabolism of VK, also known as the VK epoxide cycle, occurs in a cellular pathway, involving GGCX and VK epoxide reductase (VKOR) (55, 90). In addition, the metabolism time of different forms of VK is different. Schurgers et al. (64) found that the half-life of MK-7 was 68 h longer than 1–2 h of K_1_, leading to a higher steady serum concentration and storage of MK-7 (sevenfold to eightfold) during long-term intake. The change in the carboxylated osteocalcin/undercarboxylated osteocalcin ratio (cOC/ucOC) for MK-7 was three times greater than that for K_1_, suggesting that the higher serum concentrations of MK-7 indicated higher tissue concentrations and better utilization of MK-7 (64). As a potent antidote of oral anticoagulation, MK-7 is three to four times more effective than K_1_ (64).

## VK in Intestinal Health and Disease

### Gastrointestinal Disease Results in VK Deficiency and Will Be Further Exacerbated by VK Deficiency

VK deficiency happens in patients with fat malabsorption of any cause, attributable to intestinal injury (75), cholestatic liver disease (91), or genetic disorders (92), and the use of antibiotics (79) and anticoagulants (93). VK deficiency in the appearance of abnormal prothrombin, deficient in gamma-carboxyglutamic acid (94), may lead to serious bleeding and death (95–97). In IBD patients, VK deficiency occurs for the malabsorption resulting from intestinal damage (98). VK deficiency has also been reported in chronic gastrointestinal disorders (94), including IBDs (98–100) and short bowel syndrome (101). Actually, the levels of fat-soluble vitamins including A, D, E, and K are generally lower in patients with IBD (102). The prevalence of VK deficiency was 43.7% in UC and 54.0% in CD (75). UC and CD, as the major forms of idiopathic IBDs, are chronic inflammatory disorders of the gastrointestinal tract (103) caused by altered interactions between gut microbiome and the mucosal immune system (35). Compared with normal controls, serum VK levels of CD patients were significantly decreased (104). VK deficiency was more common in patients with higher CD activity, in CD patients with higher mass Z-scores, and less common among children with CD treated with infliximab (75). In murine models of colitis, mice fed a K-deficient diet showed more severe body weight loss, shorter colon length, and higher histological scores than those patients with IBDs fed a K-supplemented diet often exhibit VK deficiency (16). In another rat model, VK deficiency also resulted in exacerbation of murine dextran sulfate sodium (DSS)-induced colitis by IL-6 production from B cells (44). There is adequate evidence to support that VK may play a key role in the progression of CD (14), and lack of VK will exacerbate inflammatory disease.

Osteoporosis is one complication resulting from the chronic character of IBD, manifested by low bone mineral density, which leads to an increased risk of fractures (105). Malabsorption of VK is one possible factor that contributes to decreased bone mineral density (BMD), a frequent complication in gastrointestinal disease (106, 107). There is an association between VK deficiency with bone metabolism and clinical disease activity in IBD, showing that VK status and bone mineral density (BMD) are low in CD and UC patients (104). VK deficiency and decreased BMD are highly prevalent in IBD-induced osteoporosis patients, especially CD (98). VK status in patients with CD was lower than that of healthy controls, which might be an etiological factor for CD-related osteopenia (13). Lower plasma VK (K_1_ or MK-7) levels correlate with lower BMD in patients with CD and those with UC (98). Modulating the VK status may have implications for the prevention and treatment of osteoporosis in IBD (104).

### VK Has Anti-Inflammation and Immunosuppressive Function in the Intestine

The observation that high VK status was associated with lower concentrations of inflammatory markers suggests that a possible protective role by VK in inflammation merits further investigation (108). VK deficiency is seen in gut diseases, and VK-deficient conditions exacerbate gastrointestinal diseases (16, 44). Supplementation of VK showed different efficacy levels of immunosuppressive and anti-inflammation effects in *in vitro* and *in vivo* experiments of different patients and animals. On top of that, according to several safety assessments of K_2_ and K_1_ on animals and clinical and non-clinical studies together with the results of investigations conducted by reputable bodies (i.e., the EFSA, WHO the UK EVM, and the IOM), no negative effects of high-dose VK (K_1_ and K_2_) intake on animals and human beings have been found yet according to the current studies (109–113). In 2006, Ohsaki et al. (114) revealed that VK inhibited the production of IL-6 in human macrophagic THP-1 cells and that dietary supplementation of K_1_ inhibited the lipopolysaccharide (LPS)-induced inflammatory process in rats. In another *in vivo* and *in vitro* study, Ohsaki et al. further demonstrated that MK-4 exerts its effect of anti-inflammation *via* inhibiting the activation of NFκB by repressing IKKα/β phosphorylation (115). In 2016, Shiraishi et al. (16) reported that VK-deficient conditions exacerbated murine DSS colitis and that supplementation of MK-4 played an immunosuppressive role by inhibiting inflammatory cytokine production in CD19 (+) cells, for example, IL-6 and IL-10, ameliorating shorter colon length, body weight loss, and histological scores. On the other hand, a recent *in vitro* study revealed that synthetic VK (K_3_ and K_4_) rather than K_1_ and K_2_ inhibits NLRP3 inflammasome activation induced by LPS independent of the coenzyme activity and targets to block interaction between NLRP3 and ASC, hence inhibiting inflammation (116). However, the role of synthetic VK as NLRP3 inhibitor had not been verified *in vivo*, and questions on how VK blocks the NLRPS-ASC interaction and why K_2_ which could be converted from K_3_ showed no effect on activation NLRP3 inflammasome need further investigation. Although these results preliminarily demonstrated that VK had anti-inflammatory properties, huge knowledge gaps remain regarding the immunopathological effect of VK in IBD.

*In vitro* and *in vivo* experiments revealed that VK inhibited the production of pro-inflammatory cytokines, especially IL-6 and tumor necrosis factor-alpha (TNF-α) (114, 117). Administration of MK-7 showed preventive effects by suppressing CRC-risk microorganisms and metabolites (short-chain fatty acids, SCFAs), promoting serum adiponectin level, stimulating the VDR expression to trigger different anti-inflammatory and anti-tumorigenic pathways (15). K_3_, rather than K_1_ and K_2_, was reported to induce DNA damage in HT-29 human CRC cells (118). Another report showed that K_2_, K_3_, and K_5_ had efficient antitumor roles in CRC *in vivo* and *in vitro* by causing caspase-dependent apoptotic death of tumor cells (17). Supplemented VK played a safeguarding role against DSS-induced colitis and improved gut injury by suppressing inflammatory cytokine production, which could be a promising treatment target for IBDs (16). VK, as described earlier, was found to repress CRC in intensive preclinical studies. VK supplementation or deficiency, and even different sources of VK, deeply affects the intestinal status in humans and animals *in vivo* and *in vitro* (**Table 1**). Nevertheless, further studies are still required, for example, to elucidate the most effective form of VK and verify the clinical antitumor function of VK.

**Table 1:** Effects of different sources of VK on intestinal homeostasis (without bacteria information) of patients or animals *in vivo* and *in vitro*.

VK resources	Supplemented dosage	Subjects	Results	Effects	References
*In vivo*					
MK-4	75 mg kg^-1^ diet	C57BL/6 J mice of the DSS model	Body weight loss ↓ Colon length ↑ Histological scores ↓ IL-6 ↓	VK protects against DSS colitis *via* downregulating IL-6	Shiraishi et al. (16)
MK-7	50 mg kg^-1^ diet	C57BL/6J mice with DSS	Colon carcinogenesis ↓ Expression of CLCN4, p-AMVK_1_, and VDR ↑ The secretion of caecum butyric acid and acetic acid ↑	K_2_ can inhibit gut-risk microbes and increase beneficial microbial metabolites to reduce colonic tumor development in mice	Zhang et al. (15)
K_1_ or MK-4	600 mg kg^-1^ diet	Sprague–Dawley rats	IAP activity in five intestinal segments in both K_1_ and MK-4 increased ↑	Both K_1_ and K_2_ can enhance IAP activity	Sogabe et al. (119)
K_1_ and K_2_	3 mg kg^-1^ mouse	ICR strain mice	In the MK groups, the levels of ALP activity in the jejunum ↑ IAP mRNA expression in the jejunum in both K_1_ and K_2_ groups↑ The expression of pregnane X receptor mRNA ↑	Oral administration of VK enhanced IAP mRNA expression	Haraikawa et al. (120)
VK	3.02 mg kg^-1^ diet	Juvenile Jian carp	Malondialdehyde and protein carbonyl contents ↓ AHR, ASA, SOD, CAT, GST, GSH-Px, GR, activities and GSH contents in the hepatopancreas and intestine↑	VK improved fish growth, digestive and absorptive ability, and antioxidant capacity.	Yuan et al. (45)
Intravenous administration of K_2_, K_3_, and K_5_	100 mM	80-week-old male BALB/c mice	Tumor growth ↓ The number of apoptotic tumor cells ↑	K_2_, K_3_, and K_5_ played effective antitumor effects on CRC by inducing caspase-dependent apoptotic death of tumor cells.	Ogawa et al. (17)
Low K_1_	52 (control), 16, 28, 36, 49 μg kg^-1^ diet	Wistar rat	Liver K_1_ increased with the increasing K_1_ content in diet. ↑ Concentration of coagulation factors (factor II, factor V, factor VII, factor IX, factor X) in plasma. ↑ Prothrombin clotting time (s) ↓ Cecal pH ↓ Cecal wt (g), content DM (g kg^-1^) ↑ Butyrate ↑ Propionate, isobutyrate, isovalerate	The potential VK supply from enteric bacterial menaquinones may be altered by modifying diet *via* altering the density of menaquinone-producing microflora in large intestine.	Mathers et al. (121)
*In vitro*					
K_2_, K_3_, K_5_	10 mM	Colon 26, metastatic murine CRC cell line	Enzymatic activity of caspase-3 ↑	K_2_, K_3_, and K_5_ induced apoptotic death of colon 26 cells	Ogawa et al. (17)
K_1_, K_2_	200, 400, 600, 700, 800 μM K_2_; 250, 300, 400, 500, 600 μM K_1_	HT-29, human colon carcinoma cells	K_3_ caused significant DNA damage at low concentrations (25–200 μM) with a linear correlation of r 0.95	K_3_, but not K_2_ and K_1_, induced DNA damage in HT-29 human CRC cells	D’Odorico et al. (118)
MK-4	0, 1.0, 5.0, and 10.0 μM	Caco-2 cells	The ALP activities ↑ Expressions of human intestinal ALP and SI ↑	K_2_ enhanced the level of ALP mRNA expression in human Caco-2 cells	Noda et al. (46)
K_1_	10, 50, 100 and 200 μM	Human colon cancer cells (Caco-2, HT-29, SW480)	Caused inhibition of proliferation Induced apoptosis and the cell cycle arrest Enhanced the probiotic anti-proliferative effect in a dose-dependent manner ↑	K_1_ has enhanced anti-proliferative efficacy to inhibit cancer growth	Orlando et al. (122)
K_1_, K_2_, K_3_ and K_4_	5, 10 μM for K_1_ and K_2_; 1-5 μM for K_3_ and K_4_	Bone marrow-derived macrophages	IL-1β ↓ TNF-α ↓ NLRP3 inflammasome activation ↓		Zheng et al. (116)
			K_3_ and K_4_ inhibit inflammation by inactivating the NLRP3 inflammasome		

AHR, anti-hydroxyl radical; ASA, anti-superoxide anion; CAT, catalase; DSS dextran sodium sulfate; IL, interleukin; CLCN4, chloride channel-4; GR, glutathione reductase; GST, glutathione-S-transferase; GSH-Px, glutathione peroxidase; GSH, glutathione; LPS, lipopolysaccharide; SI, sucrase-isomaltase; SOD, superoxide dismutase.

↑ means increase or upregulate; ↓ represents decrease or downregulate.

### Interaction Between VK and Intestinal Microbiota as well as Microbial Metabolites

Accumulating evidence links the altered microbiota composition with the pathophysiology of IBDs (123, 124). Bacteria exert critical effects on the onset and perpetuation of gut inflammation in IBDs (125). The intestinal microorganism or bacteria present in food may produce bacterially synthesized menaquinones which contribute to K_2_ requirements in human (126). Small-intestinal bacterial overgrowth (SIBO), associated with low circulating levels of K_2_ (127), is involved in increased plasma levels of inactive MGP and results in alteration of K_2_ metabolism (128). SIBO may not increase bacterial K_2_ biosynthesis in the intestine but enhance dietary K_1_ absorption through the potentially damaged intestinal mucosa (127).The diversity of the gut microbiota was notably lower, and *Lachnospiraceae* and *Ruminococcaceae* greatly reduced in the VK-deficient group compared with the VK-normal group in a previous study (129). Compared with the VK-deficient group, supplemented with MK-4 and MK-9, reduced the relative abundance of cecal Bacteroides and Ruminococcus_1 while increased that of Lactobacillus at the genus level (130). Warfarin induced intestinal dysbiosis involving VK-expressing bacteria, which was related to the expression of VKOR (131). *Lactobacilli* exerted a pivotal part in modulating microorganisms and maintaining a microecological balance in the intestine, producing bacteriocin-like substances to suppress the overgrowth of potentially pathogenic bacteria (132). *E. coli* in the gut was known as a pathogenic bacterium with the possibility of causing enteric infection (133), while another pathogenic bacterium *Aeromonas* was associated with gastroenteritis (134). In fish, increasing levels of VK up to 3.02 mg/kg diet could enhance *Lactobacillus* (LB) but decrease *Aeromonas* and *E. coli* replications (45). The potency of VK has been proven to optimize the gut microorganisms by increasing the numbers of LB and lowering the number of *Aeromonas* and *E. coli*. In another study on rat gut, a low K_1_ level reduced the counts of health-promoting bacteria, such as *Bacteroides fragilis* and *B. vulgatus*, and enhanced the counts of pathogenic bacteria, such as *Fusobacterium*, *Bifidobacterium*, and *Enterococci*, in rat feces (121). *In vitro*, VK ameliorated the growth of the probiotics, for example, *Bifidobacterium* (135). Previous studies demonstrated that MK-7 (50 mg/kg diet) supplementation alleviated colon cancer in mice by reducing representative colonic polyps and the number of large colon tumors. The VK supplementation was effective in the enrichment of *Proteobacteria* counts, such as promoting the relative abundance of *C. lanceolatus*, *P. phenylpyruvicus*, and *Parasutterella excrementihominis* and reducing CRC-risk microbes, such as *H. mesocricetorum* and *H. apodemus* (15). Nonetheless, debates on whether all types of VK have the same beneficial effect on intestinal microbiota are ongoing (**Table 2**). Regarding the beneficial effect of VK on intestinal microflora, Ponziani et al. (128) proposed that K_2_ intake could be prescribed in clinical practice as additional preventive measures for screening SIBO and intestinal decontamination.

**Table 2:** Profile of gut microbiota after supplementation or deficiency of VK *in vivo* and effect of VK on microflora *in vitro*.

VK resources	Content of VK	Subjects	Microorganisms	References
*In vivo*				
VK-deficient	Deficient	CD patients	*Ruminococcaceae*, *Lachnospiraceae* ↓	Wagatsuma et al. (129)
VK-deficient or supplemented	VK-deficient or supplemented with 5 μmol kg^-1^ PK, MK-4, MK-7, or an equimolar combination of PK, MK-4, MK-7	Female mice of C57 BL 6J	The VK-deficient group had the lowest relative abundance of *Lactobacillus*, and the greatest relative abundances of *Bacteroides* and a *Ruminococcus genus* group (*Ruminococcus_1*).	Ellis et al. (130)
VK	3.02 mg kg^-1^	Juvenile Jian carp	LB ↑ Aeromonas, E. coli ↓	Yuan et al. (45)
Low K_1_	52 (control), 16, 28, 36, 49 μg kg^-1^ diet	Wistar rat	Bacteroides fragilis, Bacteroides vulgatus ↓ Fusobacterium, Bifidobacterium, Enterococci ↑	Mathers et al. (121)
MK-7	50 mg kg^-1^ diet	Mouse	C. lanceolatus, P. phenylpyruvicus, and Parasutterella excrementihominis ↑ H. mesocricetorum and H. apodemus ↓	Zhang et al. (15)
Diet supplemented with black-eye beans or white rice	Black-eye beans (108 μg kg^-1^ K_1_) vs. white rice (2 μg kg^-1^ K_1_)	Rat	Total Bacteroides, Bacteroides fragilis, Bacteroides vulgatus, Veilonella sp. ↑ Fusobacterium sp., Anaerobic Gram-positive rods ↓	Mathers et al. (121)
*In vitro*				
MK-4 or MK-7	5 μg ml^-1^	Bacteria were isolated from periodontally healthy subjects.	Bifidobacterium, Porphyromonas gingiva ↑	Hojo et al. (135)

↑ means increase or upregulate; ↓ represents decrease or downregulate.

Gut microbe has a variety of intestinal functions such as improving the mucosal immune system, defending against pathogens, synthesizing amino acids/vitamins, and absorbing complex macromolecules (136). Speculation on the possible underlying mechanism by which VK affects the intestinal microbiome is based on the fact that anaerobically growing bacteria, the facultatively aerobic bacteria, and most Gram-positive bacteria use MK as the sole quinone in their oxidative and photosynthetic electron transport system (137). MK inhibitors showed selective toxicity to these bacteria without any side effects due to its exclusiveness. Although VK has a toxic effect on some bacteria unrelated to the gut, the underlying mechanism of VK in the gut microflora has not been elucidated. Hence, further *in vitro* and *in vivo* investigations in the intestine are essential.

What is more, VK can alleviate IBDs by regulating microbial metabolite (SCFA) production. Microbial MK-7 could enhance the secretion of cecum acetic acid and butyric acid (15). With the increase in the K_1_ level in diet, concentrations of butyrate are enhanced and propionate, isobutyrate, and isovalerate are reduced (121). Except being used preferentially as an energy source by the enterocytes (138), microbial butyrate has the potential function to the restoration of the barrier function in IBD (139), imprint an antimicrobial program of macrophages (140), attenuate pathobiont-induced hyperinflammation (141). Propionate, capable of histone deacetylase (HDAC) inhibition, can potentiate *de novo* Treg-cell generation in the periphery (142). Acetate could promote intestinal IgA responses (143). Alterations in SCFA metabolism, particularly butyrate, occur in IBD (144). UC patients and healthy individuals have different compositions of the fecal microbiota, showing that butyrate-producing bacteria, *R hominis* and *F prausnitzii*, are reduced in UC (145). Moreover, UC has less obvious reduced butyrate-synthetic capacity of the microbiota than UC (144), while the clear relationship among VK, butyrate-producing bacteria, and butyrate remains unknown. Also, further studies trying to explain this detailed mechanism will be necessary and interesting.

### VK Against Oxidation

IBDs are associated with a disequilibrium between reactive oxygen species (ROS) and antioxidant response, giving rise to oxidative stress (146). Oxidative stress is a crucial cause in the pathophysiological process of certain chronic diseases, resulting from an imbalance between pro- and antioxidant substances (147), resulting in potential cellular damage and dysfunction (148). Several studies demonstrated oxidative stress as an important factor in the pathogenesis, progression, and severity of IBDs (146) and showed that the use of prophylactics to inhibit oxidative stress improved the health status of patients (149, 150). VK showed its ability to alleviate intestinal oxidative stress *via* regulating the expression of pro-oxidant and antioxidant enzymes (45, 151, 152).

Yuan et al. (45) conducted *in vivo* studies using dietary VK (3.13 mg/kg diet) to improve the antioxidant capabilities of digestive organs by decreasing the contents of protein carbonyl and malondialdehyde (MDA) and improving anti-hydroxyl radical (AHR), anti-superoxide anion (ASA), superoxide dismutase (SOD), glutathione (GSH), glutathione peroxidase (GSH-Px), glutathione-S-transferase (GST), catalase (CAT), and glutathione reductase (GR) activities and contents in the intestine. Nevertheless, this was a study conducted on the carp, which could not be simply extrapolated to mammals. More investigations in mammals should be performed in the future to verify the effect and mechanism of VK on related oxidoreductase activity in the intestine.

In *in vitro* studies, VK has antecedently been reported to possess free radical-scavenging activity when assayed in non-aqueous solvents (153). Studies of cell lines outside of the intestinal cells showed that the biological activity of MK-4 dose-dependently suppressed the upregulation in the expression of iNOS, COX-2, p38 activation, NF-κB, ROS, and caspase-1 activation (4) and prevented ROS from inducing oxidative damage *via* inactivating the p38 MAP kinase pathway (3, 154, 155). The disproportionate accumulation of ROS might, however, alter several cellular proteins and upregulate pro-inflammatory cytokines, further downregulating the expression of TJ proteins and triggering the deterioration of the intestinal permeability (156). It was speculated that VK might exert the same ability to prevent oxidative damage in intestinal cells *in vivo*, which needs empirical studies for validation. However, the mechanism underlying the VK protective function remains unclear to date. Thus, further analysis of its antioxidant functions in the intestine is necessary.

### VK Contributes to Blood Coagulation in Gastrointestinal Disease

GIB, due to peptic ulcer, colitis, hemorrhoids, cancer, malignancy, esophageal varices, or other conditions, occurs from upper and lower GIB (157). VK deficiency in newborns also results in massive GIB (158). Besides, GIB is a frequent and potentially serious complication of oral anticoagulant (159). The risk of GIB and subsequent complications is considerably lower for patients on non-VK antagonist oral anticoagulants (NOACs) than for patients on warfarin (160). The case fatality proportion is nearly 10% and 3% for hemorrhage of the upper and lower gastrointestinal tracts, respectively (161, 162). The rapid onset of VK deficiency in patients occurs may be due to a combination of major abdominal surgery in patients who are receiving antibiotics and poor food intake (163). GIB due to VK deficiency in patients on antibiotics usually stopped by timely injections of VK (47).

### VK and Gut Epithelial Development

Nutrient availability is closely involved in digestive and absorptive ability, which depends on the growth and development of the pancreas and intestine, and the activities of digestive enzymes such as amylase, lipase, and protease, and gut enzymes, such as IAP and sucrase-isomaltase (SI) (164). IAP, a brush-border protein, is a defense factor in the gut mucosa (165) and an intestinal crypt-villus differentiation marker at the brush border of gut epithelial cells that can detoxify LPS by dephosphorylation (46, 166). SI is a brush border enzyme of small bowel to metabolize sucrose, whose deficient condition causes symptoms of maldigestion syndromes including diarrhea, bloating, abdominal pain, and gas (167). *In vitro*, K_2_ enhances IAP and the expression of SI and may enhance the cellular differentiation and functions of Caco-2 cells (46). *In vivo*, dietary K_1_ or K_2_ (3 mg/kg mouse) supplementation enhances the activity and mRNA expression of IAP in rats and mice (119, 120). Both K_1_ and K_2_ (600 mg/kg diet) exhibited increased IAP activity in each segment of the small intestine when the small intestine of Sprague-Dawley rats was divided into five segments (119). A study proved that VK increased the IAP activity (119) by the steroid and xenobiotic receptor (SXR) in a rat model (168). MK-4 is a ligand for SXR (known as its murine ortholog, pregnane X receptor, PXR) (168–170), and PXR is abundantly expressed in the intestine and liver in mammals (171); its activation suppresses the NFκB signal pathway and relieves the severity of IBD, indicating the fundamental role for PXR in IBD treatment (172, 173). It could be speculated that VK may exert a positive role in gut *via* PXR.

VDR, regulating 1α, 25-dihydroxy vitamin D3 [1,25(OH)2D3], is richly expressed in the small bowel and colon (174), while its expression decreases in both UC and CD patients (175) and downregulated by TNF-α associated with IBD (176). VDR deficiency in the gut leads to abnormal paneth cells and impaired autophagy function, imbalance of autophagy and apoptosis in the intestinal epithelium (177), change in the function of microbiome (178), enhancement of Wnt/β-catenin signaling, and tumor burden (179). Gut VDR exerts significantly regulatory effects on immunity, anti-inflammation, cell proliferation, autophagy activation, differentiation, barrier function and permeability, and host-microbial interactions (180, 181). VK deficiency significantly increases the VDR binding to DNA and that binding was sharply reduced when gut endogenous containing VDR undergo VK-dependent gamma-carboxylation (182). In the presence of K_1_, VDR can undergo γ-carboxylation *in vitro* and that 15%-25% of Glu residues in the VDR are carboxylated *in vivo* (183). AMPK is also known to improve epithelial differentiation and barrier function, integrity, and ultrastructure of tight junction in the gut (184, 185). Vitamin D3 and the AMPK agonist metformin were observed to play synergistic preventive roles against colon cancer (186). MK-7 was found to stimulate VDR and AMPK expression effectively (15). MK-7 may have indirect potential clinical application in preventing and treating IBD by vitamin D/VDR and AMPK signaling.

ADPN is an adipocytokine, exerting anti-carcinogenic roles in colon tumorigenesis (187, 188), confirmed as a potential and promising target for CRC therapy for its anti-tumorigenic effects (189, 190). However, MK-7 interventions can elevate the expression of ADPN in rats with CRC (15). To date, emerging studies suggested substantial beneficial effects of VK on intestinal growth and function by mediating the activity and mRNA expression of IAP, ADPN, VDR, and AMPK signaling.

Even though a few studies showed promoting roles of gut epithelial development of VK, indicating potential preventive and therapeutic effects of CRC, a body of animal experiments and cell tests is in urgent need.

### VK Exerts Gastroprotection Role *via* Related VKDPs

VK is an essential cofactor of GGCX for the posttranslational conversion of peptide-bound Glu to Gla (54). VKDPs are known to be a functional protein family with Gla residues, which result from a γ-carboxylation of Glu residues and a posttranslation modification dependent of VK, and catalyzed by γ-glutamylcarboxylase (191–193) (**Figure 4**). After carboxylation, the propeptide which is essential for Gla proteins binding to the vitamin-K-dependent carboxylase is removed and the mature protein is secreted (42, 194). Among 17 kinds of recognized γ-carboxylated proteins, the biofunction of VKDPs in the intestine, such as protein C (195), protein S (196), Gas 6 (197), and MGP (198), is another speculated mechanism through which VK might relieve symptoms of gastrointestinal disease.

Thromboembolism is caused by an imbalance of procoagulant, anticoagulant, and fibrinolytic factors (199). It is an extra-intestinal manifestation and a crucial cause of morbidity and mortality in IBD (200). IBD in hypercoagulability is mainly manifested as microthrombus formation and microcirculation disorder (201), and the thrombus formation rate is between 1.2% and 7.1% (202). Protein C (PC), synthesized by the liver, is a vitamin-K-dependent glycoprotein and a natural anticoagulant protein. The PC system, playing crucial roles in anticoagulation and inflammation, is a novel participant in the pathogenesis of acute and chronic inflammatory diseases, such as IBDs (203). The defective PC pathway in both inactive and non-active diseases may result in hypercoagulability in IBD, which is associated with both the inflammatory process and disturbances in the anticoagulant system (204). In the UC mouse, the PC system is inhibited *via* the secretion of cytokines from macrophages, subsequently influencing the function of endothelial cells (195), while it could be reversed by blocking CXCR4 (205). In addition to its anticoagulant activity, the PC pathway, acting on the endothelial compartment and controlling gut homeostasis by reducing cytokine production and inhibiting leukocyte adhesion (206, 207), exerts cytoprotective effects in the gut (207, 208). Consequently, activated PC treatment can diminish weight loss (206, 207), reduce the disease activity index (207), relieve the pathological lesions (206), and reduce histological colitis scores (207). However, functionally inactive molecules of VKDPs are produced at their site of synthesis and released into the bloodstream when the supply of VK is deficient or abnormal (209). VK supplementation therapy might become a new direction in the pathogenesis and treatment of IBD *via* the activated PC pathway, and this speculation needs scientific experimental verification.

Protein S, a well-defined VK-dependent cofactor for activated protein C, exists in a free anticoagulantly active form and in an inactive form complexed to C4b-binding protein in normal adult plasma (210). Protein S can activate TAM receptors (Tyro3, Axl, and Mer) which have important effects on hemostasis and inflammation (211). It is found that the impairment of the protein S/protein C/thrombomodulin system in CD patients contributes to coagulation and might be vital for both the development of CD and its thromboembolic complications (196), while CD is mediated by multifocal gastrointestinal infarction (212) which is due to thrombosis in small vessels (196). Free plasma protein S levels are slightly but significantly decreased in IBD patients (213). Consequently, low Protein S levels are considered as a potential etiologic factor in patients with IBD and recurrent deep venous thrombosis (DVT) (214).

Gas 6 is a γ-carboxyglutamic acid domain-containing protein and a VK-dependent growth factor for mesangial and epithelial cells (215), which shares 43% amino acid identity with protein S. Gas6 is another VKDP activator of TAM receptors (211). It suppresses the production of TNF-α which is an inflammatory cytokine induced by TLR 3, 4, and 9 *via* activating TAM receptors (216). In patients with advanced colorectal cancer, the immunoreactivity of Gas6 in cancer tissues was positively associated with prognosis (197). Gas6 suppresses the progression of intestinal tumors induced by DSS correlated with inhibition of stromal immune reactions *in vivo* (197). In a great scale of human gastric cancer tissue and cell lines, there is a high expression of mRNA and protein of Gas6 (217). With recombinant Gas6 and a decoy receptor of Axl *in vitro*, the Gas6-Axl signaling pathway improved invasion and inhibited apoptosis *via* the Akt signaling pathway (217).

MGP is a kind of secreted protein, also a small Gla VKDP, and acts as a powerful naturally occurring inhibitor of calcification and has strong affinity for calcium ions (218). Its inactive form, dephosphorylated-uncarboxylated MGP (dp-ucMGP), has been regarded as one of the best markers representing low K_2_ status (219). MGP has to undergo VK-dependent carboxylation and phosphorylation to become biologically active (220). Consequently, VK deficiency leads to the inactive dp-ucMGP (220). Experimental data of a cross-sectional study in UC and CD patients support the immunomodulatory effect of MGP in IBD and involvement in the pathophysiology of the disease (221). Compared to the healthy control group, plasma levels of dp-ucMGP were significantly higher in IBD patients and positively correlated with high sensitivity C-reactive protein (hsCRP) levels (221). The expression of MGP, which can be upregulated by a conserved binding site for Egr-1 in the upstream region of the human MGP gene, was positively correlated with disease severity of UC patients and DSS-induced colitis rats (222). MGP was upregulated in different stages of colon cancer and associated with a worse prognosis (223). Endogenous MGP promotes the growth and proliferation of colon cancer cells by increasing the intracellular calcium level and activating the NF-κB pathway (223), while supplementation of exogenous mesenchymal stromal cell (MSC)-derived MGP might be a novel important mediator of MSC-mediated immunomodulation in treating CD by alleviating the clinical and histopathological severity of colonic inflammation in mouse experimental colitis models to a remarkable degree (198). Moreover, MSC-derived MGP alleviated the clinical and histopathological severity of colonic inflammation in mouse experimental colitis models to a remarkable degree (198). In another report, SIBO is associated with reduced matrix Gla-protein activation (128). *In vitro*, MSC-derived MGP was observed to suppress cell proliferation and cytokine production in T cells obviously (198), and it could serve as a potential prognostic biomarker in colon cancer patients (223).

Studies analyzed above examining the association between related VKDPs and intestinal diseases do not differentiate between the total and undercarboxylated forms or take into consideration VK intake. Consequently, a great deal of studies need to investigate the relationship between VK and the responding effects of VKDPs on the intestine.

## Conclusions and Future Perspective

**Figure 5:**
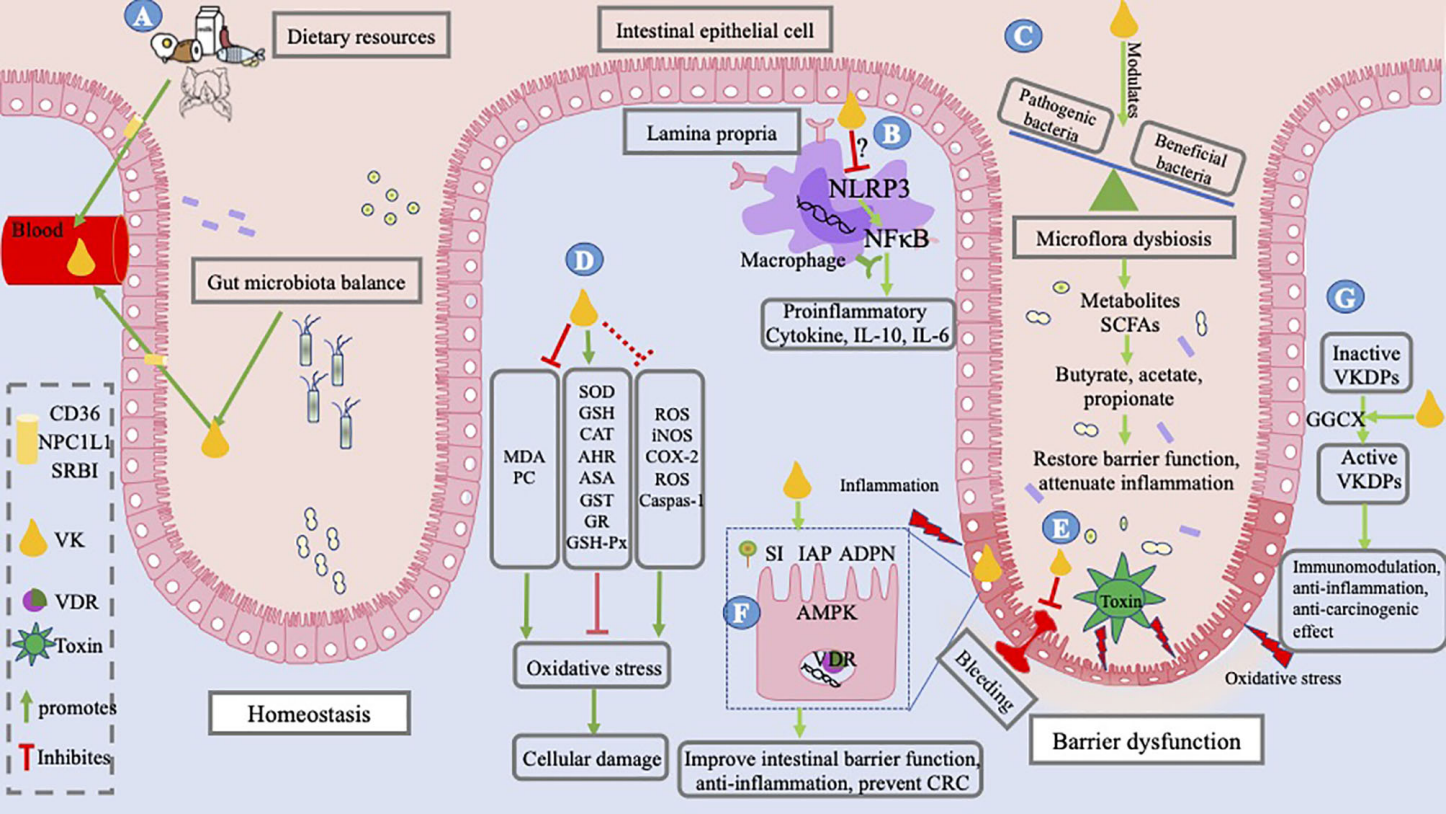
Mechanism underlying IBD and role of VK. IBD occurs as a result of altered interactions between the mucosal immune system and gut bacteria, resulting in bleeding, an imbalance between pro- and antioxidant substances, and barrier dysfunction. Toxins released by pathogenic bacteria; oxidative stress caused by oxidizers, that is, MDA, PC, and ROS; and pro-inflammatory factors induce barrier dysfunction. **(A)** VK in the body, coming from dietary resources and bacterial sources, is absorbed into the intestinal lumen; it is absorbed by small-intestinal enterocytes through the NPCIL1 protein, SR-BI, and CD 36. VK exerts a gut-protective role by alleviating intestinal inflammation and oxidation, optimizing intestinal microflora, and improving key biological enzymes in the intestine. **(B)** It may achieve an immunosuppressive function by inhibiting NLRP3 activation, thereby decreasing the inflammatory cytokine production, for example, IL-6, IL-10, and TNF-α. **(C)** VK modulates the profile of gut bacteria by inhibiting pathogenic bacteria and upregulating beneficial bacteria, thus reducing the production of toxins and regulating microbial metabolites. **(D)** VK is reported to alleviate oxidative stress and cellular damage by decreasing the levels of MDA and PC and increasing the levels of SOD, GSH, AHR, CAT, ASA, GST, GR, and GSH-Px *in vivo*, while studies of preventing ROS, iNOS, COX-2, and caspase-1 *in vitro* of cell lines outside of the intestinal cells need verification in enterocytes. **(E)** VK deficiency results in GIB and VK administration can stop GIB. **(F)** VK enhances the biological function of the intestinal epithelial cells by increasing the expression of AMPK and VDR, and intestinal enzymes, such as IAP, SI, and ADPN. **(G)** VK is essential for the activation of VKDPs and exerts indirect roles of immunomodulation, anti-inflammation, and anti-carcinogenic effects *via* VKDPs. The figure is in a non-editable format.

Coagulation has been the canonical function of VK since its discovery in 1936. The research and development studies during an over 80-year span further enhanced the benefits derived from VK. In recent years, VK has been well recognized in health and disease conditions such as type 2 diabetes mellitus, osteoporosis, CKD, cardiovascular disease, and certain cancers. Based on the present studies and publications, the direct and indirect gastrointestinal protection effects of vitamin are summarized in **Figure 5**. Novel direct functions of VK are associated with alleviating intestinal inflammation and oxidation, improving intestinal microbiota, regulating microbial metabolites, and improving epithelial development in the intestine. Indirect roles of VK are involved in anti-inflammation, immunomodulation, and anti-tumorigenesis in the gut based on the presence of certain related VKDPs. In summary, the role of VK in the improvement of gut integrity has made it a potentially useful prophylactic compound for the prevention and clinical treatment of intestinal diseases, especially for IBD. Although VK may be a potential and promising treatment target for IBD, the mechanism underlying the influence of VK on the microbial community, immunity, intestinal barrier, and antioxidation remains unknown. Scientific research on the dose–response effects of VK may be a way forward, and long-term clinical trials are necessary for confirmation in future studies.

## Author Contributions

The authors’ contributions were as follows. YL writes and BZ designed this review; the others were responsible for the modification and correction. All authors contributed to the article and approved the submitted version.

## Funding

This work was supported by the National Natural Science Foundation of China (No. 32072750) and the 2115 Talent Development Program of China Agricultural University.

## Conflict of Interest

The authors declare that the research was conducted in the absence of any commercial or financial relationships that could be construed as a potential conflict of interest.

## Publisher’s Note

All claims expressed in this article are solely those of the authors and do not necessarily represent those of their affiliated organizations, or those of the publisher, the editors and the reviewers. Any product that may be evaluated in this article, or claim that may be made by its manufacturer, is not guaranteed or endorsed by the publisher.

## Glossary

**Table:**

AHR	anti-hydroxyl radical
AMP	adenosine 5&prime;-monophosphate
AMPK	the AMP-activated protein kinase
APDN	adiponectin
ASA	anti-superoxide anion
BMD	bone mineral density
CAT	catalase
CD	Crohn&rsquo;s disease
CD 36	the cluster-determinant 36
CKD	chronic kidney disease
CLCN4	chloride channel-4
CM	chylomicron
cOC/ucOC	the carboxylated osteocalcin/undercarboxylated osteocalcin ratio
CR	chylomicron remnant
CRC	colorectal cancer
dp-ucMGP	dephosphorylated-uncarboxylated
DSS	dextran sodium sulfate
DVT	venous thrombosis
GAS6	growth arrest-specific protein 6
GGCX	γ-glutamyl carboxylase
GIB	gastrointestinal bleeding
Gla	γ-carboxyglutamate
Glu	glutamate
GSH	glutathione
GSH-Px	glutathione peroxidase
GR	glutathione reductase
GST	glutathione-S-transferase
HDAC	histone deacetylase
hsCRP	high sensitivity C-reactive protein
IAP	intestinal alkaline phosphatase
IBDs	inflammatory bowel diseases
K_1_	vitamin K_1_
K_2_	vitamin K_2_
LB	*Lactobacillus*
LPS	lipopolysaccharide
MDA	malondialdehyde
MGP	matrix Gla protein
MK	menaquinones
MSCs	Mesenchymal stromal cells
NPCIL1	the Niemann–Pick C1-like 1
NOACs	non-VK antagonist oral anticoagulants
PC	protein C
PRGP	proline-rich Gla proteins
1,25(OH)2D31α	25-dihydroxyvitamin D3
ROS	reactive oxygen species
SI	sucrase-isomaltase
SIBO	small-intestinal bacterial overgrowth
SCFAs	short-chain fatty acids
SOD	superoxide dismutase
SR-BI	the scavenger receptor class B-type I
TG	triglyceride
TMG	transmembrane Gla proteins
TNF-α	tumor necrosis factor-alpha
UBIAD1	the UbiA prenyltransferase domain-containing protein 1
UC	ulcerative colitis
VK	vitamin K
VDR	the nuclear receptor vitamin D receptor
VKDPs	VK-dependent proteins
VKO	VK epoxide
VKOR	VK epoxide reductase
